# Current Narrative Review—Application of Blood Flow Restriction Exercise in Clinical Knee Problems

**DOI:** 10.3390/medicina61081377

**Published:** 2025-07-30

**Authors:** Saehim Kwon, Ki-Cheor Bae, Chang-Jin Yon, Du-Han Kim

**Affiliations:** 1 Department of Orthopaedic Surgery, Konkuk University Medical Center, Konkuk University School of Medicine, Seoul 05030, Republic of Korea; kwonjamming@gmail.com; 2 Department of Orthopedic Surgery, Keimyung University Dongsan Hospital, Keimyung University School of Medicine, Daegu 42601, Republic of Korea; bkc@dsmc.or.kr (K.-C.B.); poweryon@dsmc.or.kr (C.-J.Y.)

**Keywords:** blood flow restriction, exercise, quadriceps muscle, anterior cruciate ligament reconstruction, knee osteoarthritis

## Abstract

Quadricep weakness is frequently observed in patients following anterior cruciate ligament (ACL) injury or in those with knee osteoarthritis, often contributing to functional impairments and persistent symptoms. While high-intensity resistance training has been shown to effectively improve muscle strength, its application may be limited in certain populations due to pain or the risk of surgical complications. In recent years, blood flow restriction (BFR) training has emerged as a promising alternative. Growing evidence indicates that low-load BFR exercise can significantly improve muscle strength, induce hypertrophy, and enhance knee function, with outcomes comparable to those of high-intensity resistance training. When implemented using appropriate protocols, BFR training appears to be a safe and efficacious rehabilitation strategy for individuals with knee pathology.

## 1. Introduction

Quadricep atrophy is commonly observed in patients undergoing knee surgery or those with knee osteoarthritis, often leading to long-term complications such as abnormal gait patterns, persistent pain, and even surgical failure [1,2,3,4,5]. Resistance training is a primary intervention to prevent or mitigate quadricep atrophy, with exercise intensity playing a critical role. It is generally accepted that loads exceeding 60–70% of one repetition maximum (1RM) are required to improve muscle strength, while 70–85% of 1RM is necessary to induce hypertrophy. However, high-intensity (HI) resistance training is often contraindicated in individuals with knee osteoarthritis or in post-operative patients due to pain, joint irritation, or the need to protect healing tissues [6,7,8,9]. Moreover, the mechanical load associated with HI training may surpass the joint’s tolerable limits, making it unsuitable during the early phases of rehabilitation. To address these limitations, various alternative modalities—such as neuromuscular electrical stimulation, blood flow restriction (BFR) training, and biofeedback-guided exercise—have been explored [10,11]. Among these, BFR training has recently garnered attention as a viable and strategic rehabilitation approach.

**Figure 1:**
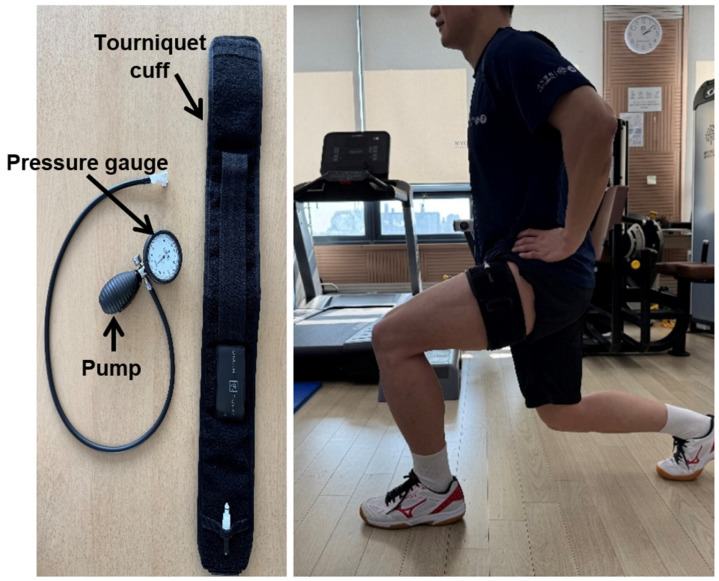
Blood flow restriction instruments consist of a tourniquet cuff, blood pressure gauge, and pump. The tourniquet cuff is used to partially restrict arterial inflow and fully restrict venous outflow during exercise. The blood pressure gauge is used to measure the pressure within the cuff. The pump is used to inflate the tourniquet cuff to the desired pressure.

BFR training, also referred to as occlusion training, was first developed by Dr. Sato in Japan in 2004 [12,13]. This technique involves the application of a pressure cuff or elastic band to the proximal portion of a limb, resulting in partial restriction of arterial inflow and complete occlusion of venous outflow during exercise. The typical equipment used for BFR training includes a tourniquet cuff, a manometer, and a manual or automatic pump (Figure 1) [6]. Despite growing interest in its potential benefits, there remains a lack of well-organized clinical research regarding the standardized application protocols and long-term outcomes of BFR training.

This narrative review aims to summarize and elucidate recent advancements in BFR training, with a particular emphasis on its application in knee-related pathologies. The review is structured as follows: (1) an overview of the fundamental principles and application techniques of BFR training; (2) an evaluation of its safety profile; and (3) a discussion of its analgesic effects, which are critical for assessing clinical feasibility. Subsequently, we review clinical outcomes in two major patient populations—individuals with (4) anterior cruciate ligament (ACL) injuries and those with (5) knee osteoarthritis—where BFR training is being increasingly utilized. Based on the current body of evidence, we then outline (6) practical application strategies commonly employed in clinical settings and conclude with (7) a discussion of limitations and directions for future research.

## 2. Principle and Application Methods of Blood Flow Restriction Training

The principle of BFR devices is to restrict blood flow to the muscles, thereby creating a hypoxic environment within the tissues and inducing temporary ischemia in the exercised area [14]. This hypoxic condition generates metabolic stress, which activates the mammalian target of the rapamycin signaling pathway responsible for muscle protein synthesis and anabolism [15,16]. This process stimulates the production of several growth factors, such as insulin-like growth factor and growth hormone, which promote muscle fiber recruitment and muscle hypertrophy [17]. In addition, BFR training activates vasoactive metabolites and vascular endothelial growth factor, thereby stimulating angiogenesis and eventually increasing post-obstructive blood flow to the limb [18].

**Figure 2:**
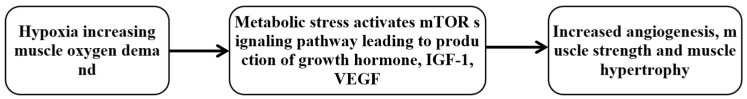
The physiological processes of the blood flow restriction exercise.

These effects are systemic, leading to strength gains and muscle hypertrophy not only in the proximal and distal musculature of the tourniquet site but also in the contralateral limb muscles [19]. The level of muscle protein synthesis and activation of the mammalian target of rapamycin pathway achieved through BFR training is comparable to that observed with HI resistance training [20]. Furthermore, signaling pathways involving protein tyrosine kinase may help inhibit mitochondrial permeability transition pores and reduce the overall concentration of reactive oxygen species, thereby minimizing cellular damage and supporting the metabolic demands of skeletal muscle, which in turn improves exercise performance capacity [15]. These mechanisms may also reduce pain and inflammation associated with HI training, thus enhancing the effectiveness of BFR training in functional recovery programs for postoperative patients or individuals with osteoarthritis [21] (Figure 2).

When implementing BFR training, specific parameters such as the degree of restriction pressure, cuff width, duration of restriction, and rest intervals vary across studies. The restriction pressure has been reported as either a fixed value of 160–200 mm Hg [9,22,23,24,25], or as a relative value set at approximately 60–80% of the arterial occlusion pressure measured using Doppler ultrasound [6,8,26,27,28,29]. Cuff widths have ranged from 6 to 20 cm [6,8,23,24,25,29], and rest intervals between sets have varied from 30 s to 1 min [6,8,23,24,25,26,27,28,29]. Some studies applied BFR only during exercise [7,22,25], whereas others maintained BFR continuously throughout the training session [8,9,23,24,26,27,29].

Due to these methodological variations, recent efforts have been made to establish standardized guidelines to enhance both the safety and efficacy of BFR training. Patterson et al. [6] proposed that, given individual differences in arterial occlusion pressure, applying restriction at 40% to 80% of the measured pressure is the safest and most appropriate approach. In a clinical trial, Mattocks et al. [30] divided participants into groups with occlusion pressures of 0%, 10%, 20%, 30%, 50%, and 90%, and found that while higher restriction pressure increased cardiovascular responses such as enhanced blood flow, it also elevated exercise intensity to a level that reduced total training volume and diminished hypertrophic adaptations.

Conversely, Hughes et al. [31] compared low-intensity (LI) exercise under low (40%) and high (80%) arterial occlusion pressures and found significantly greater pain reduction in the high-pressure group. Neto et al. [32] examined the effects of continuous versus intermittent BFR training on maximal voluntary strength (isometric and dynamic), muscle hypertrophy, and muscular endurance. While strength and hypertrophy outcomes were similar between groups, the continuous BFR group demonstrated superior gains in muscular endurance. Nielsen et al. [33] reported no muscle damage when BFR was applied at 100 mm Hg with high-frequency exercise to voluntary failure, using 30 s rest intervals and continuous restriction. Lastly, Mouser et al. [34] concluded that when restriction pressure is standardized based on arterial occlusion, variations in cuff width have minimal impact on training outcomes.

## 3. Safety of Blood Flow Restriction Training

Potential complications associated with BFR include cardiovascular responses, limb ischemia, and thrombosis [35]. Patterson et al. [18] reported that fluctuations in peripheral hemodynamics may occur following LI BFR exercise. However, Clark et al. [36] found no significant changes in pulse wave velocity or ankle-brachial index, both of which are indicators of arterial stiffness. Takano et al. [37] demonstrated that although systolic blood pressure and heart rate increased in BFR compared to non-BFR, there were no statistically significant changes in total peripheral resistance index or cardiac output, and stroke volume was significantly decreased in BFR due to reduced preload. These findings suggest that BFR does not impose a greater cardiovascular or hemodynamic risk compared to non-BFR exercise.

Furthermore, the degree of blood pressure elevation induced by LI BFR was comparable to that seen with HI non-BFR, indicating no additional risk [38]. Clark et al. [36] also conducted a study in 16 young adults who performed BFR training for four weeks, reporting no significant increases in D-dimer, high-sensitivity C-reactive protein (hs-CRP), or prothrombin time, thereby supporting the safety of BFR with respect to venous thromboembolism.

Tennent et al. [39] investigated 17 patients aged 18–65 years who underwent knee arthroscopy and were divided into BFR (80% arterial occlusion pressure) and non-BFR exercise groups. No thrombus formation was observed in either group. Calatayud et al. [40] studied eight patients with hemophilia and found that low-load resistance training with BFR at 40% of arterial occlusion pressure resulted in no adverse effects such as bleeding, and no significant differences in pain, rate of perceived exertion (RPE), or tolerability compared to the control group.

Madarame et al. [41] reported that in patients with stable ischemic heart disease who were not on anticoagulants, BFR led to significant increases in heart rate and plasma noradrenaline levels, but no significant changes were observed in D-dimer, fibrin degradation products, or hs-CRP, suggesting that BFR may be relatively safe in this population. However, due to the cardiac load it induces, caution is warranted, and the use of BFR is contraindicated in patients with unstable ischemic heart disease.

The application of BFR is not recommended for individuals with arterial calcification, sickle cell traits, severe hypertension, heart failure, unstable ischemic heart disease, or those taking procoagulant medications. Local complications may include subcutaneous hemorrhage and transient numbness due to peripheral nerve compression, though these symptoms typically resolve over time [42]. To minimize the risk of systemic or local complications, BFR should be applied under the supervision of trained professionals, following appropriate screening and individualized assessment.

## 4. Analgesic Effects of Blood Flow Restriction Training

The results of studies investigating the analgesic effects of BFR training have been variable. In patients who have undergone anterior cruciate ligament (ACL) reconstruction, research indicates that the initial perception of pain and exercise intensity, as measured by the rating of perceived exertion, in the LI BFR group is comparable to or even higher than that in the HI non-BFR group [38,43]. This heightened pain sensitivity is believed to result from the hypoxic environment within the muscle and the accumulation of lactic acid, which stimulate the release of algogenic substances such as substance P, bradykinin, histamine, and prostaglandins.

However, Martín-Hernández et al. [44] reported that although the perceived exercise intensity was similar between the LI BFR and HI non-BFR groups at the onset of training, it significantly decreased in the LI BFR group over the course of repeated sessions. In addition, anterior knee pain was found to be lower in the BFR group. Hughes et al. [31] compared four groups—low-intensity, high-intensity, low-intensity low BFR (40% arterial occlusion pressure, 30% of one-repetition maximum), and low-intensity high BFR (80% arterial occlusion pressure, 30% of one-repetition maximum)—and observed increased pressure pain thresholds and exercise-induced hypoalgesia in both BFR groups. Furthermore, the BFR groups maintained a reduced level of exercise-induced pain 24 h after the training session.

In studies by Segal et al. [23,24] involving patients with knee osteoarthritis, the LI BFR group demonstrated significantly greater reductions in pain following a 12-week exercise program compared to the non-BFR group.

## 5. Application and Clinical Outcomes of Blood Flow Restriction Training in Patients with Anterior Cruciate Ligament Injuries

Most patients who undergo ACL reconstruction exhibit quadricep weakness and muscle atrophy, which can persist for a significant period after surgery [45,46]. This condition has been associated with an increased risk of re-injury and residual instability [47]. To address this issue, BFR training after ACL reconstruction has demonstrated improvements in strength and hypertrophy of the quadriceps, hamstrings, and adductor muscles in most studies [6,22,25,28,48].

Research indicates that BFR applied for more than three months is clinically effective (Table 1). For instance, a study by Ohta et al. [22] evaluated 44 young men and women who underwent ACL reconstruction, comparing a BFR exercise group with a non-BFR exercise group over a three-month period. The results showed significant improvements in maximal strength during knee extension and flexion, as well as in the cross sectional area of the quadriceps, hamstrings, and adductor muscles in the BFR group. Muscle biopsies assessing type 1 and type 2 muscle fiber diameters in eight patients per group revealed an increase in fiber diameter in the BFR group, although the difference was not statistically significant. The Cochrane risk of bias for each study is summarized in Table 2.

Additionally, Hughes et al. [6] investigated the effects of exercise following ACL reconstruction by dividing 24 young men and women into a LI BFR exercise group and a HI non-BFR exercise group. The authors compared outcomes including muscle strength, muscle mass, knee range of motion, physical function scores, and pain levels. The LI BFR group showed improvements in strength and muscle mass that were comparable to those observed in the HI non-BFR group. Moreover, the LI BFR group demonstrated greater gains in knee range of motion, higher physical function scores, and lower pain levels [6].

## 6. Application and Clinical Outcomes of Blood Flow Restriction Training in Patients with Knee Osteoarthritis

Quadriceps weakness and thigh muscle atrophy are recognized as significant contributors to the development and progression of knee osteoarthritis [1]. In particular, as the radiographic severity of osteoarthritis advances, quadricep strength has been shown to be closely associated with the performance of daily activities and overall functional capacity [49]. Numerous studies applying BFR training in patients with knee osteoarthritis have reported improvements in muscle strength and mass, as well as enhancements in functional performance and reductions in pain levels [7,8,9].

Key studies examining the effects of BFR training in individuals with knee osteoarthritis are summarized in Table 3. Some discrepancies have been noted regarding the short-term efficacy of BFR training based on gender. Segal et al. [23,24] conducted separate studies in men and women. In a study involving 41 men aged 45 years and older with knee osteoarthritis or risk factors for osteoarthritis, a four-week intervention comparing a BFR exercise group with a non-BFR group showed no significant differences in maximal strength. However, knee clinical pain scores were higher in the BFR group. In contrast, a similar four-week study in 40 women aged 45 to 65 years with osteoarthritis or osteoarthritis risk factors demonstrated significantly greater improvements in maximal strength in the BFR group, while no significant differences in knee clinical pain scores were observed between the groups.

In contrast, most studies involving BFR applied for more than six weeks have reported clinically meaningful outcomes. For example, Ferraz et al. [8] investigated 48 women with Kellgren and Lawrence grade 2 or 3 knee osteoarthritis, who were assigned to one of three groups, LI BFR, HI non-BFR, or LI non-BFR, over a twelve-week training period. The LI BFR group demonstrated increases in maximal strength and muscle mass comparable to those observed in the HI non-BFR group, whereas no significant improvements were noted in the LI non-BFR group. Both the LI BFR and HI non-BFR groups exhibited significant gains in functional performance, including improved results on the timed stands test and enhanced knee clinical scores. Notably, only the LI BFR group showed a significant reduction in clinical stiffness. Knee clinical pain scores decreased in both the LI BFR and LI non-BFR groups, while 25% of participants in the HI non-BFR group withdrew from the study due to pain experienced during exercise. Other studies have similarly reported that the LI BFR group achieved significant improvements in muscle strength, muscle mass, and functional performance compared to the LI non-BFR group. The Cochrane risk of bias for each study is summarized in Table 4.

## 7. Current Methods and Recommendations of BFR Application Used by the Authors

**Figure 3:**
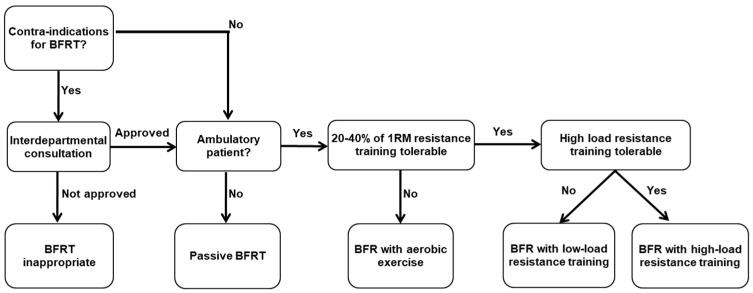
Algorithm for blood flow restriction training in a similar protocol as reported in the previous review article by Brendan R. Scott et al. [50].

In patients with ACL reconstruction or knee osteoarthritis, BFR training can be applied using a progressive algorithm (Figure 3), tailored to the individual’s ambulatory capacity and tolerance to exercise intensity.

**Figure 4:**
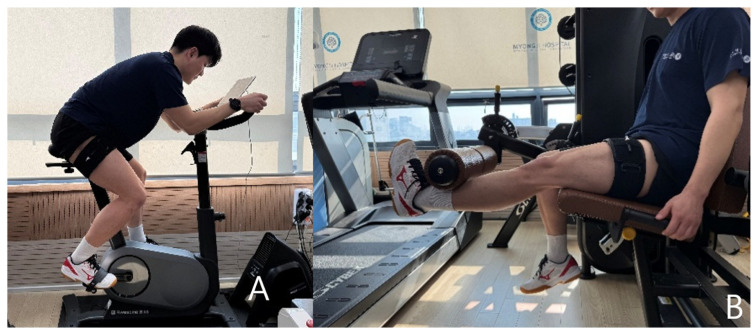
Blood flow restriction aerobic exercise (**A**) and blood flow restriction resistance training (**B**).

Passive BFR is indicated for patients who have difficulty with weight-bearing exercise or walking. When ambulation is possible, aerobic exercises such as walking or cycling are typically performed in combination with BFR (Figure 4A). BFR resistance training is introduced when the patient is able to tolerate exercise at an intensity of 20–40% of 1RM (Figure 4B).

According to current safety guidelines, BFR training is considered contraindicated in individuals with severe cardiovascular disease, uncontrolled hypertension, active thromboembolic disorders, or sickle cell anemia, unless cleared through comprehensive interdepartmental consultation. In patients with relative risk factors—such as hemophilia or stable ischemic heart disease—BFR training may still be implemented under appropriate clinical supervision, with continuous monitoring of hemodynamic responses and neurovascular status to ensure safety.

## 8. Limitations

Most of the studies cited in this review are small-scale randomized controlled trials or pilot studies, which limits the overall strength and generalizability of the current evidence. Additionally, substantial variability in BFR protocols—such as cuff pressure, cuff width, duration, and training frequency—poses challenges in establishing standardized clinical recommendations. Many studies also lack long-term follow-up and fail to incorporate functional outcome measures that accurately reflect real-world improvements in patient function. To validate the clinical efficacy of BFR in the management of knee conditions, large-scale, prospective, multicenter randomized controlled trials are warranted.

## 9. Conclusions

BFR training may serve as an effective rehabilitation strategy for patients following knee surgery or those with knee osteoarthritis who are unable to perform HI exercise and are at risk of muscle disuse. Substantial evidence supports the efficacy of low-intensity BFR training in improving muscle strength, muscular endurance, quadriceps volume, and patient-reported functional outcomes. However, findings related to performance-based functional tests and pain relief remain inconsistent across studies, highlighting the need for further investigation. Nonetheless, when applied using evidence-based protocols and maintained for an adequate duration, BFR training can provide meaningful clinical benefits while minimizing the injury risk associated with HI non-BFR exercises. Moreover, adherence to established safety guidelines reduces the risk of adverse events, including cardiovascular stress, muscle damage, thrombosis, and embolism, thereby supporting the safety and utility of BFR as a rehabilitation modality.

## Figures and Tables

**Table 1:** Outcomes of blood flow restriction exercise in anterior cruciate ligament reconstruction.

Author	Study Design	Population	Method	Outcomes
Ohta et al. [22] (2003) *	RCT	BFR (n = 22, mean age 30) -non BFR (n = 22, mean age 28)	180 mmHg, 16 weeks	-Knee extensor and flexor strength: BFR > Control CC60 86 ± 14: 84 ± 13 CC180 90 ± 9: 84 ± 14 IM60 94 ± 21: 92 ± 19 -CSA ratio of knee extensor: BFR (101 ± 11) > Control (92 ± 12)
Lambert et al. [28] † (2019)	RCT	BFR (n = 7) vs. non BFR (n = 7)	12 weeks, 20% 1RM	-Thigh lean muscle mass: BFR > Control -Similar improvements in single leg squat distance, Y-balance
Zargi et al. [25] † (2018)	RCT	BFR (n = 10) vs. non BFR (n = 10)	12 weeks, 150 mmHg, 14 cm, 30% 1RM	-Isometric endurance: BFR > control -Muscle blood flow: BFR > control
Hughes et al. [6] (2019) ^L^	RCT	BFR (n = 12) vs. non BFR (n = 12)	8 weeks -BFR: 80% LOP 11.5 cm, 30% 1RM -non BFR: 70% 1RM	-10RM and Isokinetic strength: increased in both groups (104% and 106%) -Muscle thickness: increased in both groups (5.8% and 6.7%) -Self-reported function, Quality of life and Y-balance performance(18–59 > 18–33): BFR > HL-RT -Joint pain and effusion: HL-RT > BFR
Vieira de Melo et al. [29] (2022) ^L^	RCT	BFR (n = 12) vs. non BFR (n = 12)	12 weeks -BFR: LOP 80% 30% 1RM -non BFR: 70% 1RM	-Isometric strength: BFR > control -Lysholm, IKDC and KOOS questionnaires: BFR > control -Quality of life improvement: BFR > control
Karampampa et al. [27] (2023) †	RCT	BFR (n = 8) vs. non BFR (n = 8)	12–18 w -BFR: LOP 60–80% 10–20% 1RM -non BFR: 10–20% 1RM	Thigh circumference: BFR > control Isokinetic strength: BFR (13.4) > control (9.6) No significant difference in quality of life
Robert A Jack 2nd et al. [26] (2023) ^L^	RCT	BFR (n = 17) vs. non BFR (n = 15)	12 weeks -BFR: LOP 80% 20% 1RM -non BFR: 20% 1RM	LE-LM: BFR (−0.39) > control (−4.67) LE bone mass: BFR (−2.58) > control (−16.95) Similar improvement in single-leg squat, single-leg eccentric step-down, Y-balance Return to sports time: BFR group < control

RCT; randomized control study; BFR; blood flow restriction; CSA; cross sectional area; EMG; electromyography; LOP; limb occlusion pressure; HL-RT; heavy-load resistance training; IKDC; the international knee documentation committee; KOOS; the knee injury and osteoarthritis outcome; LE; lower extremity; LM; lean mass. Risk of bias: high risk *; some concern †; low risk ^L^.

**Table 2:** Cochrane risk of bias 2.

	Randomization Process	Deviations from Intended Interventions	Measurement of the Outcome	Missing Outcome Data	Selection of the Reported Result	Overall Risk of Bias
Ohta et al. [22] (2003)	Some concerns	High risk	High risk	Low risk	Low risk	High risk
Lambert et al. [28] (2019)	Low risk	Some concerns	Low risk	Low risk	Low risk	Some concerns
Zargi et al. [25] (2018)	Some concerns	High risk	Some concerns	Low risk	Low risk	Some concerns
Hughes et al. [6] (2019)	Low risk	Some concerns	Low risk	Low risk	Low risk	Low risk
Vieira de Melo et al. [29] (2022)	Low risk	Low risk	Low risk	Low risk	Low risk	Low risk
Karampampa et al. [27] (2023)	Low risk	Some concerns	Low risk	Low risk	Low risk	Some concerns
Robert A Jack 2nd et al. [26] (2023)	Low risk	Low risk	Low risk	Low risk	Low risk	Low risk

**Table 3:** Outcomes of blood flow restriction exercise in osteoarthritis patients.

Author	Study Design	Population	Method	Outcomes
Segal et al. [23] (2015) ^L^	RCT	BFR (n = 19, 58.4 years) vs. non BFR (n = 22, 56.1 years)	160–200 mmHg, 4 weeks 30% 1RM	-Bilateral leg press 1RM: Increased significantly in both groups -Isokinetic strength: BFR (3.1) < control (4.7) -KOOS scores: BFR (4.9) < control (14.2)
Segal et al. [24] (2015) ^L^	RCT	BFR (n = 19) vs. non BFR (n = 21)	160–200 mmHg, 4 weeks 30% 1RM	-Bilateral leg press 1RM: BFR (28.3) > control (15.6) -Isokinetic strength: BFR (0.07) > control (−0.05) -Quadriceps volume: no significant group differences -Knee pain: no significant group differences
Bryk et al. [9] (2016) †	RCT	BFR (n = 17) vs. non BFR (n = 17)	6 weeks -BFR: 200 mmHg 30% 1RM -non BFR: Pressure not described; 70% 1RM	-Improved strength, function, pain in both groups -Anterior knee discomfort: BFR < control
Ferraz et al. [8] (2018) †	RCT	BFR with LIRT (n = 16, 60.7 years) non BFR HI-RT (n = 16, 59.9 years) non BFR LI-RT (n = 16, 60.3 years)	12 weeks -BFR with LIRT: LOP 70%, 20–30% 1RM -non BFR HI-RT: 50–80% 1RM -non BFR LI-RT: 20–30% 1RM	-Significant leg press (26% and 33%) and knee extension 1RM (23% and 22%) increase in BFR and HI-RT but not in LI-RT -Quadriceps CSA (7% and 8%) increase in BFR and HI-RT but not in LI-RT -TST improvement in HI-RT and BFR but not significant with TUG -WOMAC physical function (−42% and −49%) improved in HI-RT and BFRT -WOMAC pain (−49% and −42%) improved in BFRT and LI-RT -No difference in quality of life among the three groups
Harper et al. [7] (2019) ^L^	RCT	BFR (n = 19) vs. non BFR (n = 16)	12 weeks -BFR: [Pressure = 0.5 × SBP + 2(thigh circumference) + 5] 20% 1RM -non BFR MI-RT: 60% 1RM	-Isokinetic strength: improvement in both groups -Function (400 m walk, SPPB, LLFDI) improvement in both groups -Pain (WOMAC and NPRS) improvement in both groups -No significant difference in Serum P3NP, TWEAK, IGF-1 levels

RCT; randomized control study; BFR; blood flow restriction; KOOS; the knee injury and osteoarthritis outcome; LI-RT; low-intensity resistance training; HI-RT; high-intensity resistance training; CSA; cross sectional area; TST; Timed stands test; TUG; timed up and go test; WOMAC; the Western Ontario and McMaster universities osteoarthritis index; MI-RT; moderate-intensity resistance training; SPPB; short physical performance battery; LLFDI; late life function and disability instrument; NPRS; numeric pain rating scale; P3NP; N-terminal peptide of procollagen type III; TWEAK; tumor necrosis-like weak inducer of apoptosis; IGF-1; insulin-like growth factor. Risk of bias: some concern †; low risk ^L^.

**Table 4:** Cochrane risk of bias 2.

	Randomization Process	Deviations from Intended Interventions	Measurement of the Outcome	Missing Outcome Data	Selection of the Reported Result	Overall Risk of Bias
Segal et al. [23] (2015)	Low risk	Low risk	Low risk	Low risk	Low risk	Low risk
Segal et al. [24] (2015, Men)	Low risk	Low risk	Low risk	Low risk	Low risk	Low risk
Harper et al. [7] (2019)	Low risk	Some concerns	Low risk	Low risk	Low risk	Some concerns
Bryk et al. [9] (2016)	Low risk	Some concerns	Low risk	Low risk	Low risk	Some concerns
Ferraz et al. [8] (2018)	Low risk	Low risk	Low risk	Low risk	Low risk	Low risk

## Data Availability

Data sharing is not applicable. No new data were created or analyzed in this study.
